# The Contribution of Head Movement to the Externalization and Internalization of Sounds

**DOI:** 10.1371/journal.pone.0083068

**Published:** 2013-12-02

**Authors:** W. Owen Brimijoin, Alan W. Boyd, Michael A. Akeroyd

**Affiliations:** MRC Institute of Hearing Research (Scottish Section), Glasgow, United Kingdom; Baycrest Hospital, Canada

## Abstract

**Background:**

When stimuli are presented over headphones, they are typically perceived as internalized; i.e., they appear to emanate from inside the head. Sounds presented in the free-field tend to be externalized, i.e., perceived to be emanating from a source in the world. This phenomenon is frequently attributed to reverberation and to the spectral characteristics of the sounds: those sounds whose spectrum and reverberation matches that of free-field signals arriving at the ear canal tend to be more frequently externalized. Another factor, however, is that the virtual location of signals presented over headphones moves in perfect concert with any movements of the head, whereas the location of free-field signals moves in opposition to head movements. The effects of head movement have not been systematically disentangled from reverberation and/or spectral cues, so we measured the degree to which movements contribute to externalization.

**Methodology/Principal Findings:**

We performed two experiments: 1) Using motion tracking and free-field loudspeaker presentation, we presented signals that moved in their spatial location to match listeners’ head movements. 2) Using motion tracking and binaural room impulse responses, we presented filtered signals over headphones that appeared to remain static relative to the world. The results from experiment 1 showed that free-field signals from the front that move with the head are less likely to be externalized (23%) than those that remain fixed (63%). Experiment 2 showed that virtual signals whose position was fixed relative to the world are more likely to be externalized (65%) than those fixed relative to the head (20%), regardless of the fidelity of the individual impulse responses.

**Conclusions/Significance:**

Head movements play a significant role in the externalization of sound sources. These findings imply tight integration between binaural cues and self motion cues and underscore the importance of self motion for spatial auditory perception.

## Introduction

An externalized sound is one appearing to originate from a source out in the world, whereas an internalized sound appears to originate from somewhere inside the head [1,2]. Sounds presented over headphones are typically internalized, whereas real-world signals tend to be externalized. This difference is often attributed to the spectral attributes of the signal [3,4], the amount of reverberation present in the signal [5,6], and/or the way in which the signal source appears to move with the head [7-9].

### The role of spectral cues in externalization

The pinna acts as a directionally dependent spectral filter, producing prominent peaks and notches in a free-field sound’s spectrum that vary as a function of source position [10]. In contrast, sounds presented over headphones bypass the filtering properties of the head and ears. The resulting lack of any spectral cues to source direction is thought to contribute to a partial collapse of externalization. The spectral filtering properties of the head and ears as well as any room reverberation may be captured in a measurement known as a binaural room impulse response (BRIR). An anechoically recorded signal convolved with a perfectly measured BRIR and played over spectrally corrected headphones ought to be perceived as externalized because if the BRIR is spectrally accurate and also captures binaural difference cues in their entirety, the acoustics of the convolved signal at both ear canals would precisely match that of a real external sound. That is, a sufficiently accurate synthetic reproduction of the temporal and spectral features of natural external sounds should be indistinguishable from reality. 

A practical difficulty with this postulate is that it is arguably impossible to define a perfect BRIR. Errors can result from a wide variety of sources [11]: the frequency response and position of in-ear microphones, the mechanics of presentation of the signals used to measure the impulse responses, the variable resonances of the cavity produced by headphones placed over the ear, and listener movement, to name but a few. Because of these difficulties, work has been done investigating what features of a BRIR are necessary and sufficient for externalization [3,4]. That is, one goal of research is to purposely use *imperfect* BRIRs to establish what minimal spectral fidelity is required to still produce a realistic percept. 

### The role of direct to reverberant ratio in externalization

A second cue that has been implicated in the phenomenon of externalization is that of direct-to-reverberant ratio [5,6]. The argument is that reverberation provides a sense of depth to the acoustic environment. Distance (depth) perception and externalization could be said to be inextricably linked with one another, since a signal could not be perceived as being external if its perceived distance from the head is zero. Since it contains no relevant real-world reverberation, a sound presented over headphones is unlikely to have originated from out in the world. Experimental data supports the assertion that signals presented anechoically are less frequently externalized than those presented in more typical reverberant environments [12,13].

### The role of head movements in externalization

Even if it were actually possible to measure a perfectly accurate BRIR and present signals with realistic reverberation, a confounding factor for externalization is that the real acoustic world is in constant motion because the head is never perfectly still. When using BRIRs under normal headphone presentation, as a listener turns, the virtual location of a signal moves with the head. This is in contrast to sounds presented in the free field; for these sounds each time the head turns, the auditory environment turns by the same amount, but in the opposite direction. This rotation causes changes in binaural cues. Path-length differences from the sound source to the two ears cause an interaural time difference that changes as a function of source (or head) angle. The head shadow effect attenuates high frequencies on the side of the head furthest from the sound source, creating an interaural *level* difference that also changes as a function of angle [10]. The dynamics of how these cues change with head movement is a viable source of information that could be used to shape perceptual judgments, but it remains unclear the perceptual weight which listeners apply to these cues in externalizing sound sources.

Head movements have long been discussed for their role in spatial perception [14]. Listeners who are free to move their heads have been repeatedly shown to be more accurate at locating a sound source than they are when their heads are fixed [15-17]. In particular, fixing the head results in a large increase in front/back confusions [18]. A direct effect of self-movement on auditory spatial localization has been shown with movable funnels [19] and head-coupled loudspeaker selectors [14]. Wallach [14] argued convincingly that head movements play a role in determining whether sounds were presented from ahead or behind a listener. Work from our lab provided evidence that such “ego-centric” auditory motion is as salient a cue in determining front from back as are spectral cues [20].

In spite of the clear relationship between head movements and spatial auditory processing, past efforts to establish the role of head movements in the spatial phenomenon of *externalization* have been inconclusive. It was hypothesized that head movement must play a role in externalization as early as the late 1800s [21] and similar postulates have been put forward periodically in the literature ever since [22-24]. However, some more recent experimental data shows that small head movements do not play a role in the externalization of signals [25]. Begault and colleagues [26], as well as Sone et al [26,27], have also suggested that motion does not play a strong role in the degree to which a sound is externalized. Others, on the other hand, have suggested that movement *is* an important factor in externalization [7-9], although quantitative data is lacking. 

In an attempt to clarify these conflicting results, we attempted to measure how much of a role of head movement plays in externalization in two separate experiments. The experiments used infrared motion tracking and 3D audio rendering to move, in real-time, the location of real and virtual signals as a function of listener head movements. Both experiments used accurate pinna-related spectral cues and reverberation cues to isolate the role of movement in externalization. Our purpose was to determine whether signals whose source appears to remain fixed with respect to the world are more likely to be externalized than acoustically identical ones whose source appears to move with the head.

### Experiment One: The internalization of free-field signals

 The first experiment was conducted in the free-field in a hemi-anechoic chamber. We tracked the listener’s heads and used motion tracking and panning between loudspeakers to move free-field signals in a 1:1 relationship with the head movement (e.g., a signal presented at +60° relative to the head remained at +60° regardless of which direction the listener turned). The externalization of these moving signals was compared to that of non-moving, statically presented signals. 

### Experiment Two: The externalization of headphone signals

The second experiment was conducted in a reverberant room and like previous work in this lab [28] made use of virtual acoustics, but it was extended to use motion tracking and real-time digital signal processing. Using interpolated sets of generic and individualized binaural impulse responses, we created signals whose source appeared to remain spatially fixed with respect to the world as a listener turned. The externalization of these perceptually stabilized signals was compared to that of signals presented normally over headphones, moving with the head as it turned. 

## Results

### Experiment One: The internalization of free-field signals

**Figure 1:**
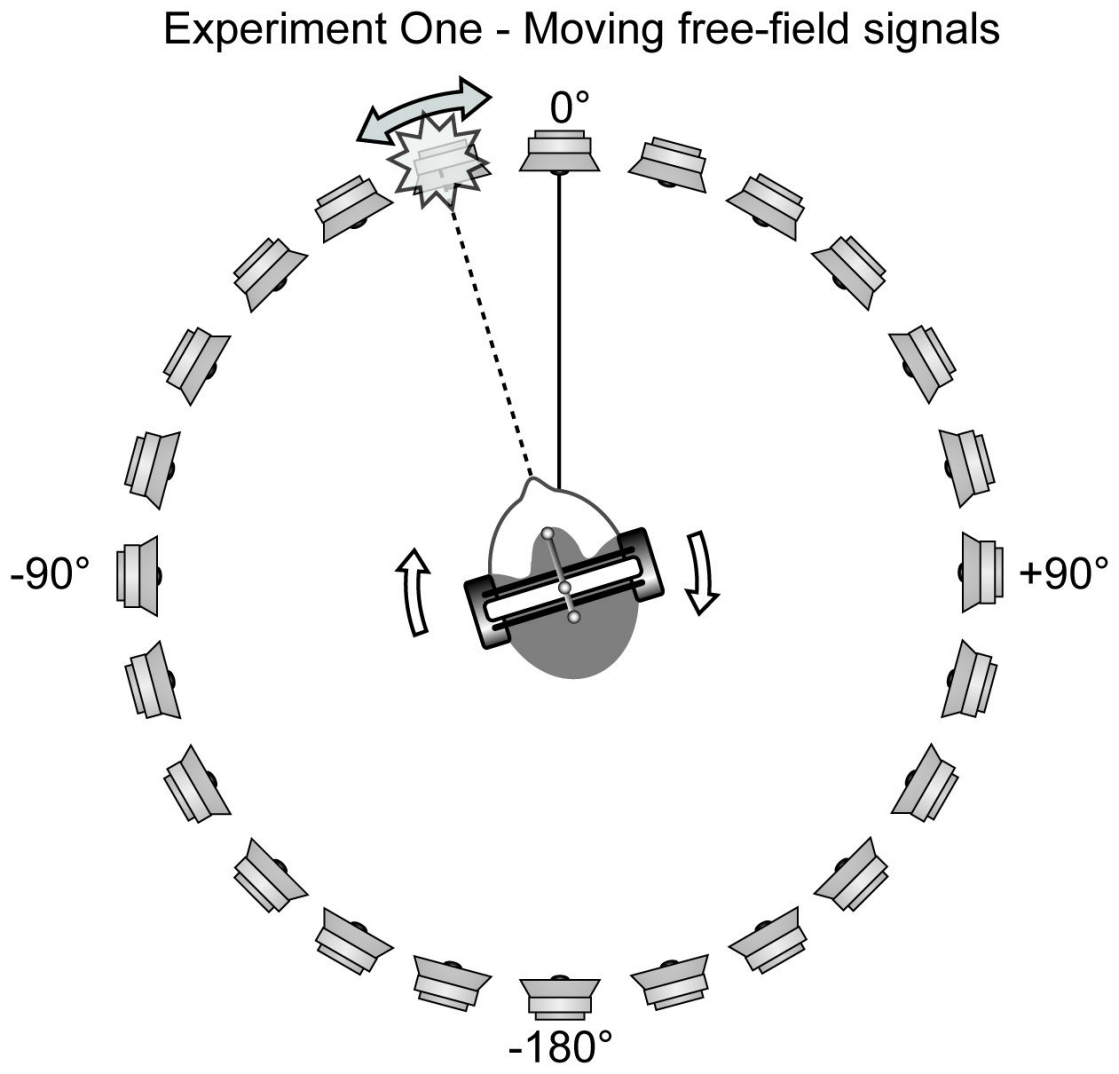
Experiment 1 methods: free-field signals moving in synchrony with the head. Signals were presented to a listener seated at the center of a free-field loudspeaker ring 1.9 m in diameter (proportions of the head are exaggerated). Using the LED array mounted on top of the listener’s head, the signals could be dynamically panned in synchrony with any head movements.

Listeners were asked to turn their heads gently back and forth between ± 15° while listening to a sequence of short sentences, and were asked to report whether each sentence emanated from either inside or outside the head. The signals were presented from a variety of angles from a ring of loudspeakers in a hemi-anechoic chamber. The signals were either presented from a particular fixed loudspeaker or were panned in real-time so as to move with the listener’s head movements (Figure 1). The experiment was run with two conditions: either with fullband signals so as to ensure that head and pinna related spectral cues and reverberation would be accessible by the listeners; or with signals lowpass filtered at 500 Hz to examine whether the elimination of high-frequency spectral cues affected externalization in our paradigm. 

#### Movement Statistics

**Figure 2:**
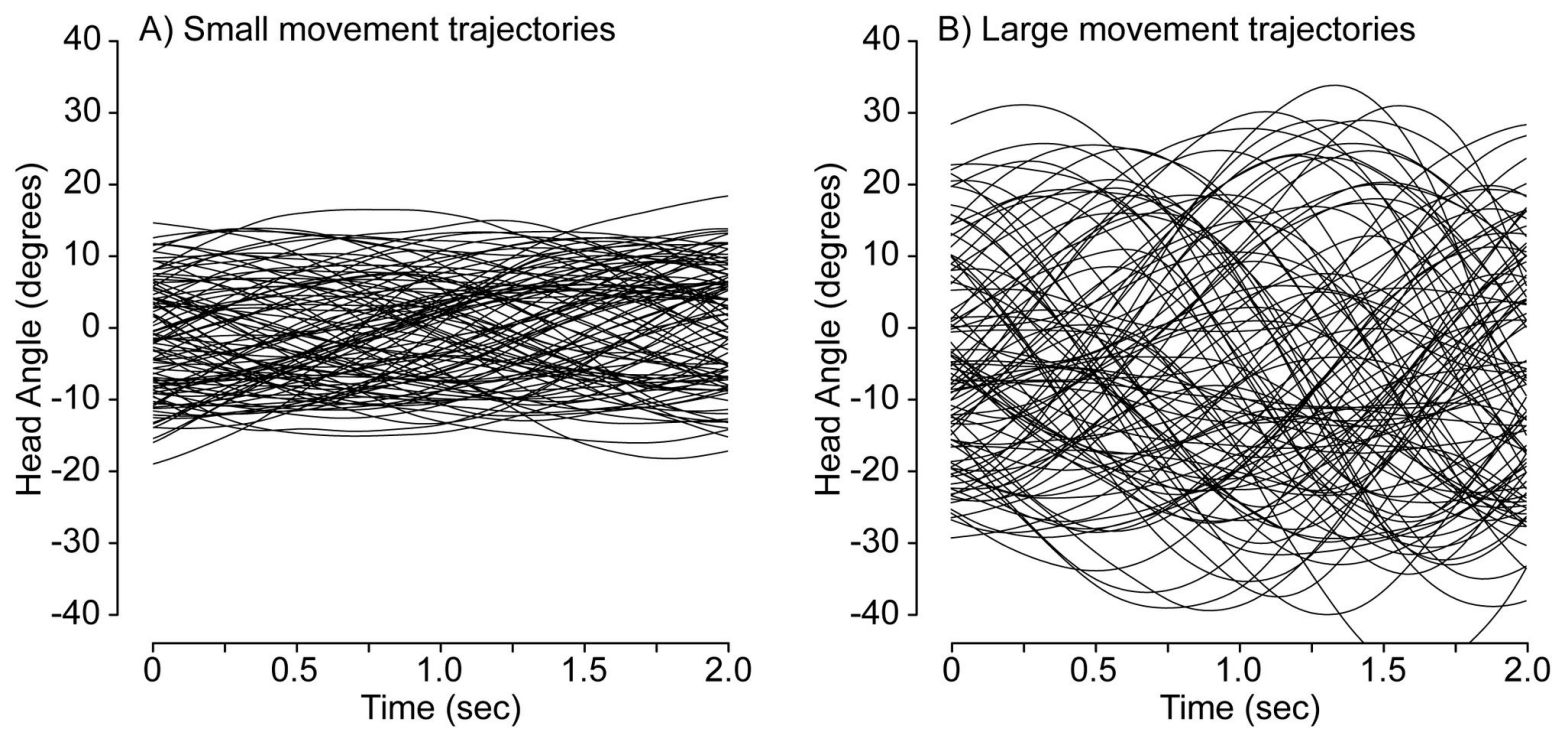
Example head movement trajectories. (A) 100 trajectories from the listener with the smallest movements. (B) 100 trajectories from the listener with the largest movements.

The movements employed by listeners varied in their extent and their velocity. Trajectories were smoothed with a 120 ms Hanning window so as to reduce the measurement noise prior to estimation of total movement and average velocity. Example trajectories from two listeners are shown in Figure 2. Sample trajectories recorded from a listener who made the smallest movements are shown in the panel on the left, while trajectories from a listener who made the largest movements are shown on the right. On average, listeners moved a total of 29° ± 12° SD during each trial (computed as the difference between the most positive and most negative head angles over the course of a given trial). The mean absolute rotational velocity during the trials was 34°/sec ± 19°/sec SD. 

#### Condition 1: Fullband signals

**Figure 3:**
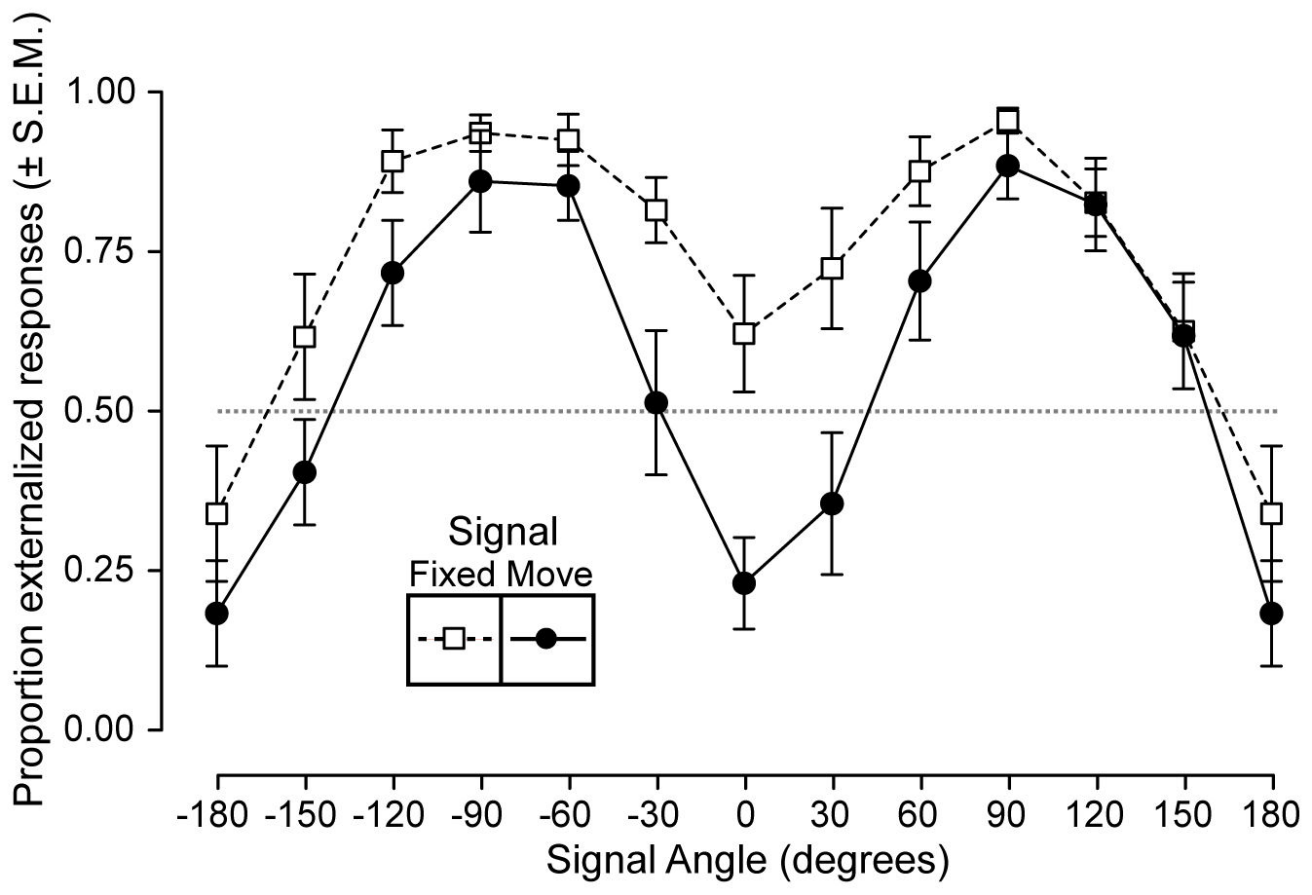
Free-field externalization results (fullband signals). The degree of externalization is plotted as a function of signal direction. The data from the signal fixed condition is plotted as open circles with dashed lines and the data from the signal moving condition is plotted as filled circles with solid lines. Note that the data points at +180° and -180° are duplicates of one another. Signals that move with the head are less likely to be externalized than those that remain fixed with respect to a particular loudspeaker, especially for signals located directly in front and behind the listener.

On the signal fixed trials (i.e., when the stimulus was simply presented from a particular loudspeaker), presentation angle strongly affected the degree to which signals were externalized (Figure 3, squares, dashed lines). Signals presented from either -90 or +90° were almost always judged to be external, in agreement with previous work [29]. Further we found that signals from either behind or directly in front of the listener were less likely to be externalized; this angle-dependent externalization is similar to that seen in previous studies using virtual acoustics [30,31]. A similar pattern was observed for the signal moving trials (Figure 3, circles, solid lines), though the overall degree to which these signals were externalized was reduced. Signals from directly ahead (0°) or behind (180°) and that tracked in their position with the listener’s movement were highly likely to be internalized. 

#### Condition 2: Lowpass filtered signals

**Figure 4:**
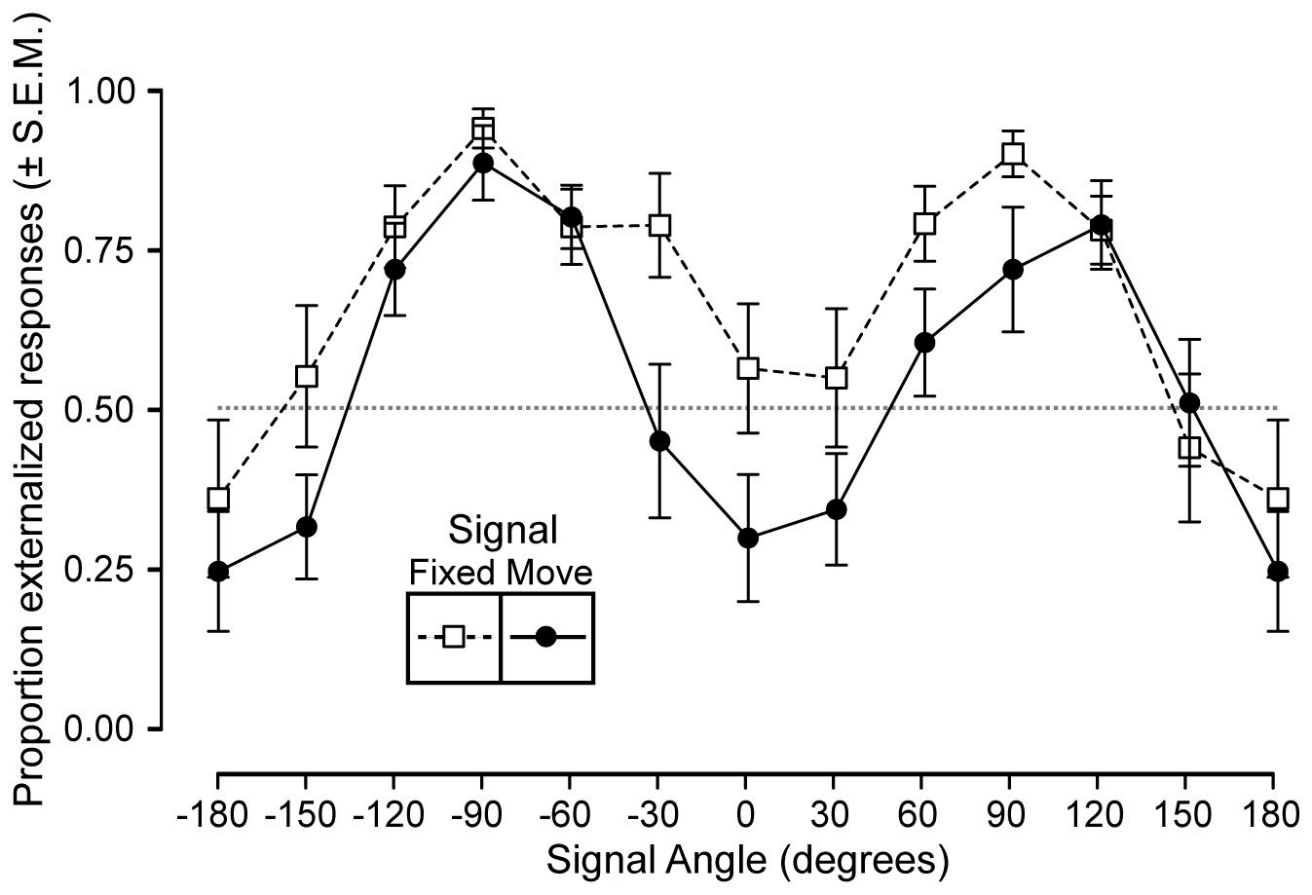
Free-field externalization results (lowpass filtered signals). The degree of externalization is plotted as a function of signal direction. The data from the signal fixed condition is plotted as open circles with dashed lines and the data from the signal moving condition is plotted as filled circles with solid lines. Note that the data points at +180° and -180° are duplicates of one another. Signals that move with the head are less likely to be externalized than those that remain fixed with respect to a particular loudspeaker.

When the stimuli were lowpass filtered at 500 Hz, a similar pattern of signal angle-dependent externalization was observed, albeit reduced overall. Signals to the right and left of the listener were more likely to be externalized than those from the front or the back (Figure 4, squares and dashed lines). Again similar to the fullband condition, signals that tracked with the head were more frequently internalized (Figure 4, circles, solid lines). The difference in the signal static / moving conditions was less pronounced than in the fullband condition. This was possibly due to the fact that lowpass filtered signals were less likely to be externalized than fullband signals. 

#### Summary Statistics

The results of a two-way ANOVA showed that presentation angle significantly affected the degree to which signals were externalized (F_(11,240)_ = 14.9, p <0.001). For free-field signals presented from all angles, the grand average proportion of externalized judgements was 71%; this dropped to 56% when the signals were panned so as to move in synchrony with the head. This main effect of signal movement condition was significant (F_(1,240)_ = 28.0, p <0 .001). If the analysis is restricted to fullband signals presented from the front, then the mean proportion of externalized judgments reduced from 63% to 23% when the signals were moved with the head. A post-hoc t-test confirmed that this difference was significant (F_(1,20)_ = 11.3, p = 0.003). The amount of listener movement was not correlated with the effect of head movement on externalization (r^2^ = 0.02). This result likely does not reflect independence of the two factors, rather it is due to the fact that the listener who moved the least nonetheless moved enough on average (~18°) to invoke some baseline level of motion. The minimum required movement to elicit an effect should be a question for future research.

### Experiment Two: The Externalization of Headphone-Presented Signals

**Figure 5:**
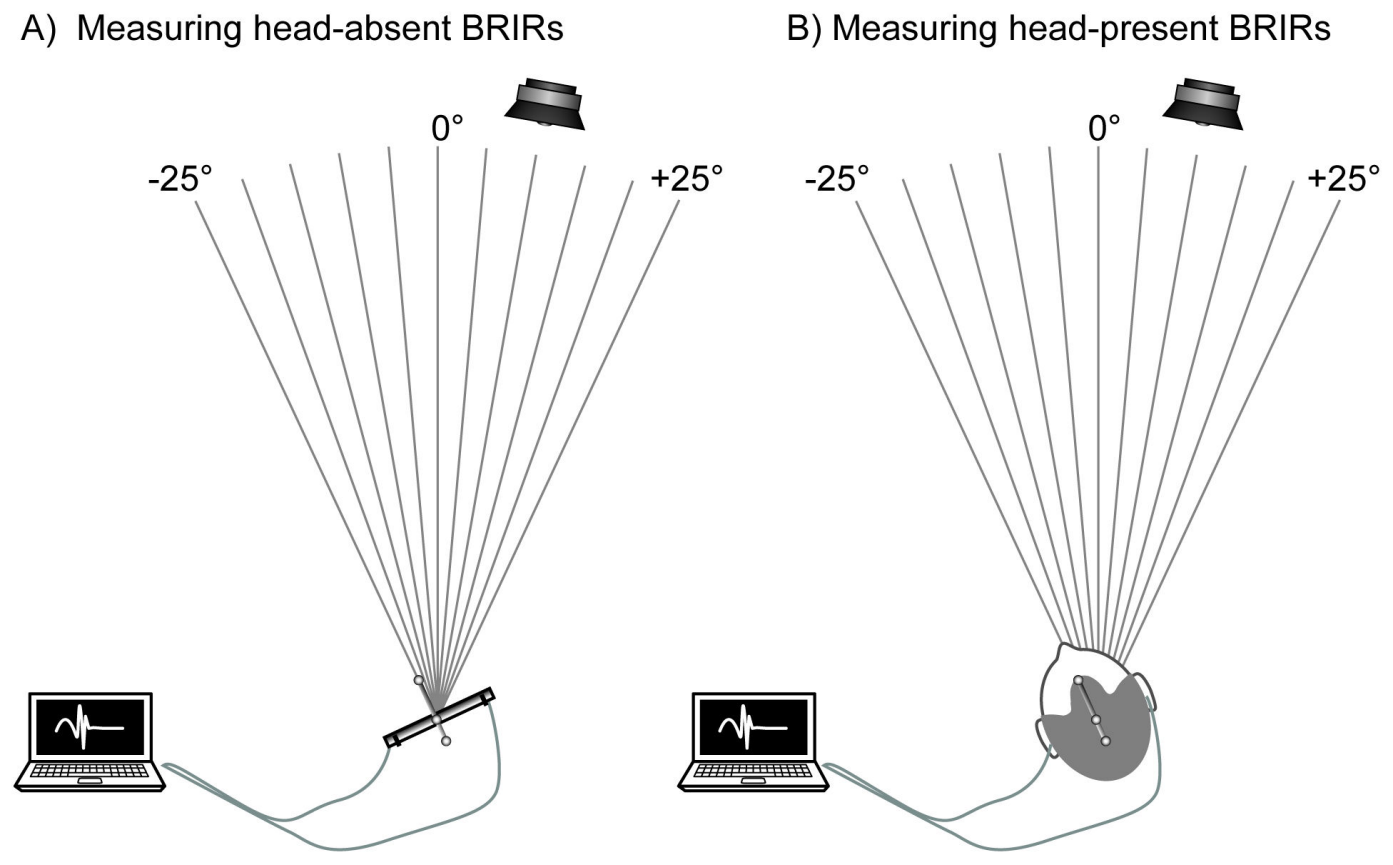
Experiment 2 methods: Measuring head-absent and head-present impulse responses. Two sets of impulse responses were recorded: head-absent impulse responses (A) were recorded with small microphones at 11 angles from -25° to +25° and head-present impulse responses (B) were recorded at the same angles with the microphones placed in the ear.

Two sets of impulse responses were measured: head-present and head-absent. Each set consisted of 11 impulse responses, recorded at angles from -25 to +25° (see Figure 5). The principle behind the measurement of the two sets was that head-present impulse responses (i.e., those captured using in-ear microphones) could be used to create virtual signals that would be reliably externalized, whereas head-absent impulse responses (i.e., those captured by a pair of microphones on a bar) should result in virtual signals that contain relevant ITD and reverberation cues but would be reliably internalized. These two sets of binaural impulse responses were mixed using linear interpolation so as to create 6 sets of hybrid impulse responses ranging from purely head-absent to purely head-present. The use of these different mixes allowed us to create a signal set that should have a gradient of externalization from inside the head to out in the world. 

#### Movement Statistics

Movement data was not archived for this experiment, making it impossible to generate quantitative summaries of listener trajectories. That said, both experiments were observed by the experimenters and the extent and velocity of listeners’ head movements in experiment two were judged to be equivalent to those seen in experiment one. 

#### Condition 1 – Head Static (Figure 6, dotted lines)

**Figure 6:**
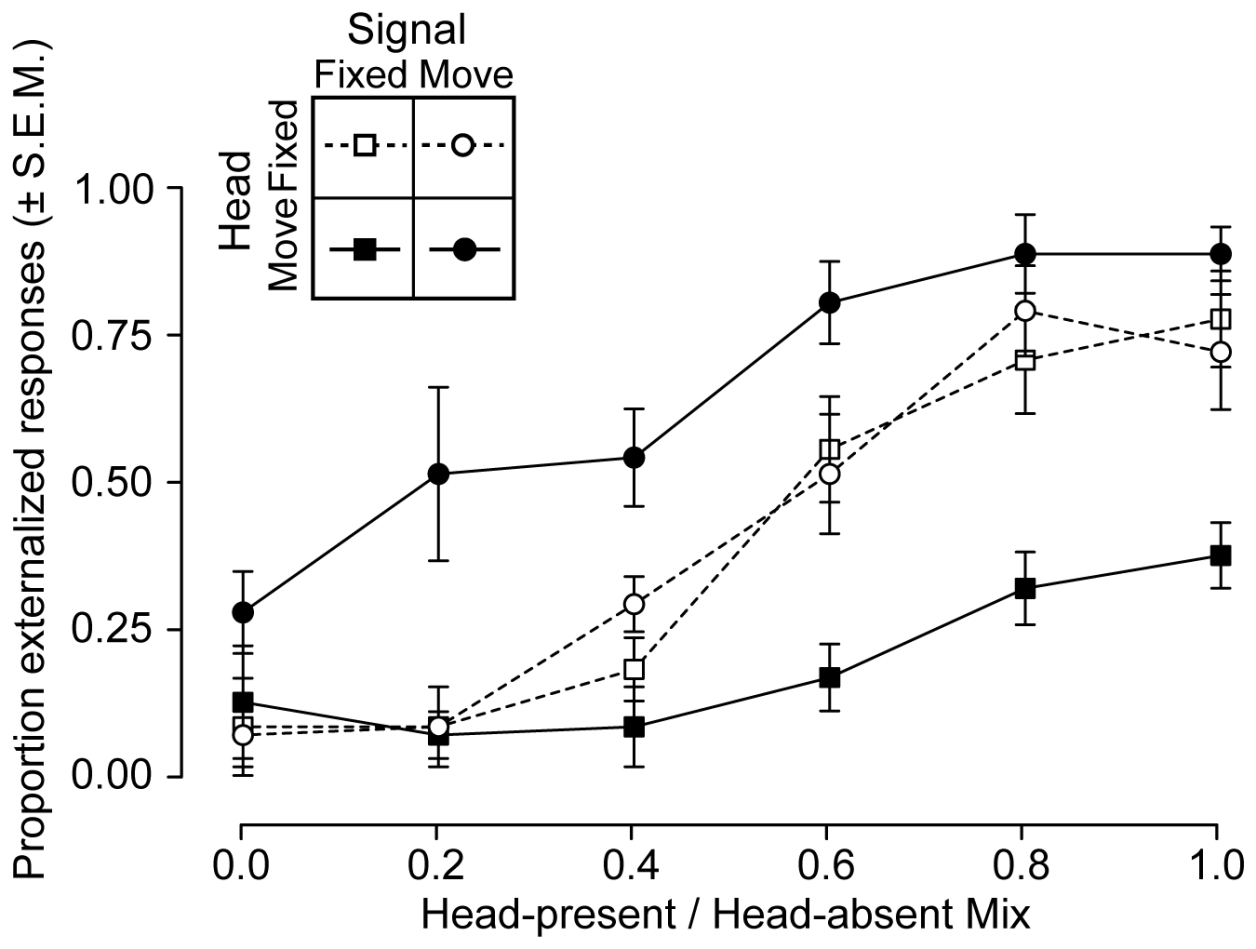
Virtual acoustics externalization results. The degree of externalization is plotted as a function of head-present / head-absent mix, varying from all head-absent (0.0) to all head-present (1.0). Head fixed conditions are plotted as dotted lines and open symbols, head moving conditions are plotted as solid lines and filled symbols. Signal fixed trials are plotted as squares and signal moving trials are plotted as circles.

Listeners were asked to remain still and report whether signals emanated from location inside or outside the head. They were presented with signals convolved with an impulse response set pseudo-randomly drawn from the 6 sets of hybrids. The results are shown in Figure 6. As the ratio of head-present to head-absent impulse response was increased from 0 to 1.0, the degree to which the resulting signals were externalized increased in a roughly sigmoidal manner (squares and dotted lines). This shows that the more individualized information the BRIR contained, the more likely listeners were to externalize the resulting signal. For the trials in which the signal appeared to remain static in space (circles and dotted lines), the same increase in externalization was seen. That these patterns of increase were essentially identical likely reflects the fact that in the head static condition it was irrelevant whether or not the signal was spatially stabilized against the listener’s movement. 

#### Condition 2 – Head Moving (Figure 6, solid lines)

When listeners were asked to turn their heads gently back and forth between ± 15°, signal-related differences in the degree of externalization emerged. For the signal-fixed condition (i.e., the one corresponding to normal headphone presentation), signals were less likely to be externalized (squares, solid lines). Note that in purely head-present condition (i.e., mix = 1.0), the proportion of time the signals were externalized was less than half of that as during the head static control condition. In contrast, when the signals were perceptually stabilized against the listener’s movement, there was a marked increase in externalization (circles, solid lines). For example, consider the case of the 0.2 mix BRIR, a signal containing only 20% of the head-present BRIR information: the proportion of trials that were externalized increased from 0.1 in the static case to 0.5 in the signal moving case. This highly spectrally-degraded BRIR nonetheless resulted in externalization in half of the trials. 

#### Summary Statistics

In the head-moving trials, for signals that remained fixed with respect to the world, across all the head-present / head-absent mix levels, signals were externalized on 65% of the trials. This was 3.5 times more frequent than when than signals were fixed relative to the head (20%). The results of a three-way ANOVA showed main effects of mix level (F_(5,120)_ = 40.2, p < 0.001) and signal movement condition (F_(1,120)_ = 56.8, p < 0.001), but no main effect of head movement condition (F_(1,120)_ = 0.26, p = .61). There were, however, significant interactions between head movement and both mix level (F_(5,120)_ = 3.4, p = 0.007) and signal movement conditions (F_(5,120)_ = 50.3, p <0 .001). 

When the all head-present mix (Figure 6, mix = 1.0) is considered in isolation, the difference between the head static and head moving conditions was not large for moving signals: 78% versus 89% (F_(1)_ = 1.4, p = 0.27) , likely due to a ceiling effect. However, during moving trials, when the signals were not spatially stabilized, the result was a large collapse in externalization (from 89% to 38%). A post-hoc t-test confirmed that this drop was significant (F_(1)_ = 50.0, p < 0.001). 

## Discussion

The question of the nature and cause of externalization of sounds has been discussed in the literature for over a century [21]. Some intriguing hypotheses have been advanced, for example that it relies on the proper ratio of bone-conducted versus air-conducted signals [27]. Shirmer [22] proposed a series of other hypotheses: that the impedance of headphone presented sounds is different from that of free-field presented signals, that the pressure of headphone pads on the ears causes problems, or that unaccounted-for transmission differences in the left and right channels result in a collapse of externalization. The modern general consensus, however, is that the ‘realism’ of the acoustics of signals entering the ear canal is critical: maintaining the correct interaural level and time differences, especially low-frequency interaural phase differences is important [1]; or more generally, if the spectral detail of free-field signals are synthesized accurately enough, the resulting percept should be that of an externalized sound, even when presented over headphones [11,32]. Reverberation certainly plays an important role, since anechoic signals are far less likely to be externalized [6,12,13]. Our data demonstrates, however, that even if the acoustic cues and room reverberation are carefully reproduced at the ear, the failure of a signal to move correctly with respect to the head can result in a large collapse of externalization.

Normal behavior ensures that movement-related cues are essentially constantly present: the head is never perfectly still even when a person is told to remain so; over several seconds the head can move in azimuth by up to 5° when unsupported [33]. When performing tasks such as watching movies or playing games, such movements can be substantially larger than this [34]. Given the ever-present nature of head movements, the phenomenon of inside-the-head versus outside-the-head sound sources is argued to be the result of a combination of the acoustic features of the auditory signal with an ongoing internal comparison between one’s own movement and the apparent movement of the sound source. We assume that the comparison takes the form of an integration of vestibular input, motor commands, and/or visual cues with smoothly-changing binaural cues, although it should be noted that present results cannot exclude the possibility that the comparison also involves smoothly-changing head-related *spectral* cues. 

The substantial role of head movement in externalization demonstrated here, while in agreement with theory and a number of previous observations, is at odds with some findings. Differences in methodology and the lack of complete information make comparing our results with those of conflicting studies problematic. For example, neither Begault et al [26] nor Sone et al [27] provided any information on the extent and velocity of their participants’ head movements. As such we cannot firmly establish that their techniques were similar enough for direct comparison. The head movements allowed in Experiment One of Toole’s study [25] were not rotational as they were in our study; rather they were translational and restricted to within 3 to 4 inches. Given that in that experiment the loudspeakers were positioned 6 feet from the subjects’ heads, this movement corresponds to a parallax-induced change in subtended angle of just over 6° at maximum: far smaller than the 30° of motion allowed in our study. In Toole’s Experiment Two, in which the loudspeakers were attached to the head, the head moving condition was restricted to natural involuntary movements that resulted from the head being unrestrained. Thus any movements that might have decreased externalization would likely have been too small to have a consistent effect. 

### Distance Perception and Externalization

Unlike some previous work from our lab which asked participants to respond on a sliding scale [28], in the current experiments (like Ohl et. al. [35]) we presented the externalization question as a binary one: “did the signal sound like it was coming from out on the world or from inside your head?” In reality, the distinction is not a binary one, but a question of degree: signals can seem to originate from points intermediate between the head and the loudspeaker, or may seem to come from locations close to the head without being perceptually located between the ears, for example. This scale is related to (and perhaps simply a semantic difference from) distance perception. At the least it is reasonable to claim that one cannot externalize a signal if it is perceived as having zero distance from the head. Very little work has been performed using motion tracking to examine distance perception (cf 36), so this is an open research question.

### Interactions between externalization and visual targets

It is reasonable to suppose that seeing a target that has a certain likelihood of being the signal source may impact externalization. The ventriloquist effect, in which the presence of a visual target can draw the perceived location of the sound source towards it, is typically described as involving a visual object and a sound source spatially separated by azimuth and/or elevation. It is a powerful effect: evidence suggests that it remains robust at audio/visual source displacements equal to or even larger than 30° [37], at least for relevant visual stimuli. For simple lights, amplitude modulated with the level fluctuations of an acoustic signal, the effect is less strong: with a 20° disparity, voices and flashing lights are perceptually fused roughly 50% of the time [38]. Our loudspeakers were visible, but were even less visually relevant than flashing lights, suggesting that any ventriloquism effect may have been minimal, especially for Experiment One in which the loudspeaker ring created many possible visual targets. 

As an aside, any ventriloquist effect invoked in our experiments may be thought of as operating not only over differences in source direction, but over differences in distance as well. In the case of the experimental manipulations that triggered an internalized percept, the way in which the signals moved and the way in which the head moved created a conflict between the absolute distance between the apparent (visual) signal source and the perceptual location of the sound – here different in azimuth and in distance.

### Summary

The results from Experiment One suggest that free-field signals that move with the head are more likely to be internalized, especially when originating from a narrow range of angles at the front of the head. The results from Experiment Two suggest that even a degraded BRIR can still evoke externalization as long as head movement is taken into account. The interrelationship of the faithful recreation of signal acoustics and the faithful recreation of movement cues has implications for synthesis of virtual audio, as well as for the processing of signals in a hearing aid. Not incidentally, many hearing aid users do report internalized sounds [39]. Thus for a device or sound reproduction system to create a believable and realistic percept of a sound emanating from a source in space, it is of benefit to take into account and balance the requirements of accurate acoustics and accurate movement. Given our results and the continuous nature of head movements, we argue that the way in which the auditory world moves as a result of head movements constitutes an ever-present, useable, and likely a *used* cue for externalization.

## Materials and Methods

### Methods common to both experiments

#### Ethics Statement

The experiment was conducted in accordance with procedures approved by the West of Scotland Research Ethics Service.

#### Listeners

Eleven listeners participated in experiment one and 6 participated in experiment two. Each listener was paid £5.00 for their participation. There was no overlap in subjects between the two experiments. All listeners had self-reported normal hearing and ranged in age from 25 to 45 years. Listeners were asked if they were experiencing tinnitus or dizziness before undertaking the experiment: no such difficulties were reported.

#### Stimuli

**Figure 7:**
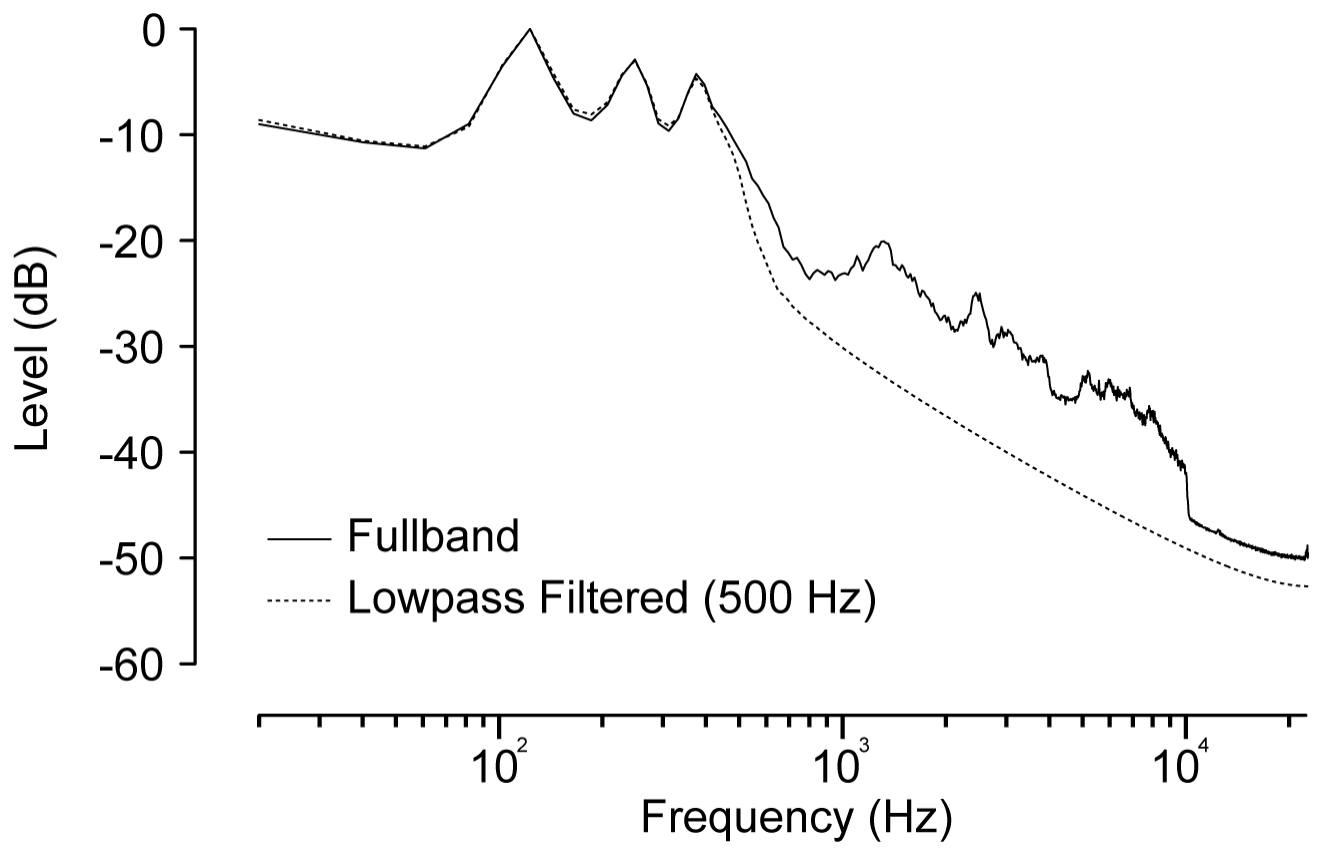
Mean spectrum of the ASL stimuli. The solid line represents the fullband spectrum and the dotted line represents the spectrum of the signals after lowpass filtering at 500 Hz.

All stimuli in both experiments were drawn randomly at run time from the Adaptive Sentence List (ASL) and consisted of short (~2 sec) sentences presented at a comfortable listening level. Sample rate of all signals was 44100 Hz. The average spectrum of the ASL sentences is shown in Figure 7.

#### Motion tracking

**Figure 8:**
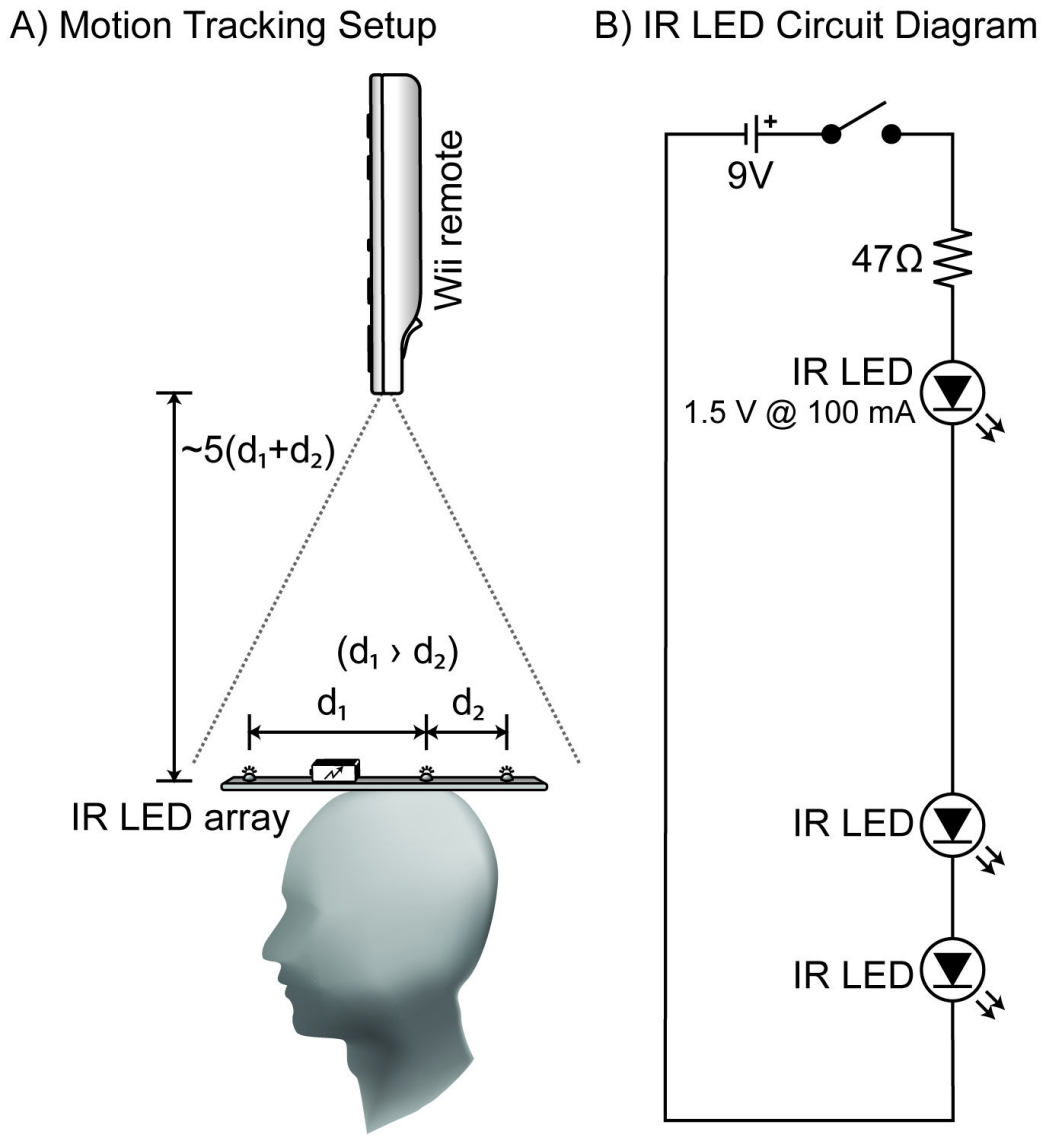
Wii remote motion tracking methods. (A) dimensions of the infrared LED array and illustration of typical tracking setup: the Wii remote was placed about 1 meter above the head pointing down at the LED array mounted on top of the listener’s head. To ensure radial asymmetry, the two LEDs at the back of the array were placed closer to each other than to the one at the front. (B) Circuit diagram illustrating the components and connections used to build the infrared LED array.

Motion tracking was performed using a Nintendo ™ Wii remote and a custom infrared LED array. The Wii remote was placed 1.5 meters above the head of the listener, with the built-in infrared camera pointing down at the top of the head. On the listener’s head was mounted a 20 centimeter long array of three infrared LEDs, powered with a 9V battery. The LEDs were arranged as shown in Figure 8, with the two rear LEDs positioned closer to each other than to the LED at the front of the array. This radially-asymmetric arrangement, assuming detection of all three LEDs, allowed an unambiguous determination of the 360° orientation of the LED array and thus the listener’s head.

The Wii remote was connected to the host PC over Bluetooth and communication was enabled by using the dynamic link library found in the WiiLAB toolbox [40]. The XY position of each of the three LEDs in the array could be polled from within Matlab at a rate of 100 Hz. Once the front and rear of the array were established by measuring the relative Euclidian distances between all the detected LEDs, an arctangent transform of the XY positions of the front and rear LEDs provided a measure of the listener’s head angle. For Matlab motion tracking code and instructions on building a simple battery-powered infrared LED array, please visit the IHR website: www.ihr.mrc.ac.uk/projects/motrack. 

#### Statistics

Statistical tests took the form of multi-way ANOVAs with alpha set to 0.05 (except in the case of the two post-hoc t-tests in condition two of experiment one, where alpha was adjusted to 0.025). The ANOVAs as well as all post-hoc t-tests were performed using SPSS version 21 (IBM, Armonk NY, USA).

### Experiment One methods

#### Room and acoustic treatment

The experiment was conducted in a hemi-anechoic chamber approximately 4 meters in all dimensions (the chamber was hemi-anechoic due to a plywood floor that covered a substantial portion of the wire mesh flooring). Extra 0.75 meter fiberglass wedges were placed around the loudspeaker stands where possible to minimize the reflectivity of the floor. The room was lit with four 60W incandescent bulbs during the experiment.

#### Sound presentation

Sound was presented via a 1.5 meter radius ring of 24 powered monitor loudspeakers (Genelec 8020B) mounted on stands. The angular position of each loudspeaker was verified to within 1° with a laser protractor. All signals were played via Matlab using three 8-channel digital-to-analog audio interfaces (MOTU 896mk3 as the host, and a MOTU Traveler mk3 and a MOTU 2408 as slaves over the AudioWire bus). The three interfaces were controlled from a single time-synchronized firewire connection and treated as a single 24-channel interface by the MOTU firewire audio console. The “playrec” library (www.playrec.co.uk) was used as a bridge between Matlab and the PortAudio API to allow real time updating of the 24-channel audio buffer.

#### Digital Signal Processing

The angular position of the signals varied across trials and, in the case of the signal-moving condition, was varied during the presentation of the sentence. To present a signal from a particular angle, it was panned between the two nearest loudspeakers using equal power panning (i.e., sine/cosine level adjustment). The sentences were broken up into chunks of 24 channels of 512 samples. The first 480 of these samples were sent to the audio buffer and the remaining 32 were held in an array for linear cross fading with the first 32 samples of the next buffer segment. At any given moment the audio pipeline contained the currently playing buffer and one or (at maximum) two cued-up buffer chunks of 480 samples. A single buffer chunk was 480/44100 = 10.9 ms in duration. This method allowed for seamless updating of the buffers during playback of the full sentences with a total update latency (time from a head turn to a change in presentation angle) ranging from a minimum latency of 22 ms to a maximum latency of 33 ms. To ensure that the processing of the signals (and thus any processing-related artifacts) in the two conditions was as similar as possible, a small amount of spatial jitter was introduced into the angular position of the signal in each buffer segment. This jitter was achieved by adding an angle randomly drawn from a uniform distribution from -0.1 to +0.1° on all trials, regardless of condition.

#### Experimental Protocol

Each listener was seated in a chair in the centre of the loudspeaker ring and fitted with a pair of circumaural headphones (Sennheiser HD 580). The headphones were modified to remove the drivers and diaphragms, but the visually-opaque inner mesh in the earpieces was left in place. This was done in an attempt to render them largely acoustically transparent (although it should be assumed that the directionally-dependent filtering properties of the pinna were to some degree affected by the remaining plastic hoop around the ear). Metal weights were added to the inside of the headphones to ensure that their weight remained the same after removal of the drivers. The headphone cable was re-attached to the inside of the headphones and run through the pass-through in the wall of the anechoic chamber. These finished “dummy” headphones were indistinguishable from a stock pair of HD 580s. This was done to ensure that the listeners could reasonably conclude that sounds could be presented from either the loudspeaker ring or the headphones. They were not told that sounds were never presented through the headphones until after the experiment was completed. 

Listeners were asked to turn their heads gently back and forth between ± 15° throughout the duration of the experiment. The infrared LED array described above was attached to the top of the headphones and a Wii remote provided the subject’s head angle 100 times every second. Listeners were presented with a single ASL sentence from a particular location in space. After the sentence was played, they were asked to press the ‘up’ button on a separate handheld Wii remote if they perceived that the signal came from out in the world and the ‘down’ button if they perceived that the signal was coming from inside their heads. In condition one [Fixed Signal], 18 sentences were presented from each of the following 12 loudspeakers: -180 ±150 ±120 ±90 ±60 ±30, and 0°, with the order of the angles randomized. In condition two [Moving Signal], the same set of angles was used, but the actual presentation angle was panned in real time to remain constant relative to the subject’s head, rather than with reference to a particular loudspeaker (for example, on the +30° trial the signal was presented from an angle of +30° relative to the listener regardless of where his/her head was pointed). The fixed and moving signal conditions were fully randomized within blocks. Two blocks of these two filtering conditions were run, one with fullband signals, and one in which the signals were lowpass filtered at 500 Hz. Each condition was presented twice, each angle was presented 12 times in the signal fixed and signal move conditions resulting in 24 measurements for each data point for each listener. 

### Experiment Two Methods

#### Room and acoustic treatment

The experiment was conducted in a large, quiet room measuring 6.5 x 5 x 3 m. The room contained three doors, two desks, three chairs, and a carpeted floor. The RT30 of the room was 0.35 seconds. The subjects were seated in a chair in the centre of the room. The room was lit with standard fluorescent office lights during the experiment.

#### Impulse response measurement

To measure both sets of head-present and head-absent impulse responses, swept sine signals were played from a JBL Control One loudspeaker located 10° to the right and 2 meters in front of the listener. Using a method similar to one used by Ohl et al [35] after Wightman and Kistler [11,32], eight concatenated swept-sine signals (20Hz-20kHz) were played from the loudspeaker in succession at 75 dBA and simultaneously recorded by in-ear microphones (The Sound Professionals MS-TFB-2). This was repeated for 11 head angles in 5° increments, varying from -25 to +25°. The listener’s head was not fixed; however, the listener’s head angle was measured using the motion tracking system immediately prior to recording and the listener was asked to keep their head still. We repeated the recordings when the head angle had changed by more than 2 degrees during measurement. For the head-absent condition the in-ear microphones were placed on a horizontal bar, 18 cm apart and at the same height as the loudspeaker (1.2 m). The same 11 angles were measured for this set. The binaural room impulse responses (BRIRs) were extracted using the technique given by Berdahl and Smith [41]. The recorded swept sine signals were cyclically deconvolved with the original and averaged, resulting in the extraction of the BRIR. In order to allow real-time convolution of the signals during the experiment, all BRIRs were truncated to 4096 samples. 

A linearly weighted mix of the head-present and head-absent impulse response sets were used to create six sets of hybrid impulse responses that were intended to vary in how likely they were to be externalized. For the weighted sum, the weights applied to the head-present impulse responses were 0.0, 0.2, 0.4, 0.6, 0.8, and 1.0, while the head-absent impulse responses were conversely weighted with 1.0, 0.8, 0.6, 0.4, 0.2 ,and 0.0. Thus the hybrid impulse response sets varied from a set consisting only of the head-absent to a set of purely head-present impulse responses. 

#### Headphone equalization

The stimuli were spectrally equalized for headphone playback by presenting the swept-sine signals over headphones and recording them through the microphones in the ear, creating headphone-equalized binaural-impulse responses for headphone playback. In the frequency domain, using the inverse of the extracted impulses for equalization could result in large peaks in the filter and small variations in the position of the headphones in relation to the microphones could vary the filter shape [42] To reduce these effects, the headphones were removed and then replaced by the participant after presentation of two swept-sine signals.

#### Sound presentation

During the experiment, randomly chosen and dynamically filtered (see below) ASL sentences were presented from a pair of AKG k702 headphones powered by an M-Audio FastTrack 8 channel USB interface. This audio interface was connected to a laptop running Matlab r14, which handled the running of the experiment and the signal processing and buffering of the audio. The loudspeaker that was used to measure the impulse responses was left in place for the testing-phase of the experiment. 

#### Digital Signal Processing

The two closest impulse responses to the listener’s current head angle (see motion tracking methods section) were chosen and linearly interpolated. This interpolation was necessary given the 5° spatial resolution of the collected impulse responses, and resulted in a perceptual approximation of intermediate source directions. Signals consisting of monaurally recorded ASL sentences were preceded by 3584 zeroes and segmented into chunks of 4096 samples that overlapped by 3584 samples. These chunks were then convolved with the interpolated binaural impulse response to yield a 2-channel signal, 4096 samples in duration. The last 512 of these samples were selected for playback. 480 of these were sent to the audio buffer and the remaining 32 were held in an array for linear cross fading with the first 32 samples of the next buffer segment. This rolling window method allowed the reverberant tail of preceding signals to be updated with the currently used impulse response. The latency from a head turn to a change in apparent source location was similar to that in experiment 1: ranging from between 22 ms to a maximum latency of 33 ms. The same spatial jitter as in experiment one was applied to ensure that processing in the conditions was analogous. 

#### Experimental Protocol

Each listener was seated in the same position in the reverberant room as they were when the impulse responses were measured and were presented with a series of filtered ASL sentences presented over headphones. After each sentence was presented, listeners were asked to press the ‘up’ button on a handheld Wii remote if they perceived the signals as having come from out in the world or the ‘down’ button if they perceived them as having originated from inside their heads.

The experiment consisted of two conditions: 1) head moving and 2) head fixed. Condition order was randomized, so that some listeners did the signal moving trial first. Within each condition, the impulse response filtering of the signal was either adjusted to compensate for any head movement (signal move) or left unadjusted so that signals were fixed in their apparent position relative to the head (signal fixed). In the head moving condition, listeners were asked to turn their heads gently back and forth between ± 15°, whereas in the head fixed condition they were asked to remain still with their heads pointed at a black dot on the wall in front of them at 0°. Condition order was randomized and within each condition, the order of [signal-move] and [signal-fixed] trials was randomized. Each impulse response was used 12 times in signal fixed and signal move trials, and each head movement condition was repeated once, allowing 24 measurements for each data point in each listener.
